# Effect of Mineral Supplementation on the Macromineral Concentration in Blood in Pre- and Postpartum Blackbelly Sheep

**DOI:** 10.3390/ani10071206

**Published:** 2020-07-16

**Authors:** Juan Carlos Moyano Tapia, Simon Alexander Leib, Pablo Roberto Marini, Maria Laura Fischman

**Affiliations:** 1 Facultad de Ciencias de la Vida/Centro de Investigación, Posgrado y Conservación Amazónica, Universidad Estatal Amazónica, Puyo 160150, Ecuador; simonleib@gmx.de; 2 Centro Latinoamericano de Estudios de Problemáticas Lecheras (CLEPL), Casilda 2170, Argentina; pmarini@unr.edu.ar (P.R.M.); fischman@fvet.uba.ar (M.L.F.); 3 Facultad de Ciencias Veterinarias, Universidad Nacional de Rosario, Casilda 2170, Argentina; 4 Consejo de Investigaciones (CIC-UNR), Rosario 2000, Argentina; 5 Facultad de Ciencias Veterinarias, Universidad de Buenos Aires, CABA 1427, Argentina; 6 Instituto de Investigación y Tecnología en Reproducción Animal (INITRA), Universidad de Buenos Aires, CABA 1427, Argentina

**Keywords:** minerals, sheep, pre- and postpartum, grazing system

## Abstract

**Simple Summary:**

The objective of this study was to determine the effect of mineral supplementation on the serum concentration of calcium, phosphorus, and magnesium in pre- and postpartum Blackbelly sheep throughout three successive lambing periods under free grazing conditions in the Ecuadorian Amazon Region. The field work was carried out between January 2015 and February 2018 using 20 Blackbelly females. The flock was randomly divided into two groups: Group 1 (G1), who were fed forage plus a supplementation (Pecutrin^®^ Mineral supplement plus vitamins A, D3, and E. Bayer HealthCare); and Group 2 (G2), who were fed forage only without mineral supplementation. In this study, we showed that Blackbelly sheep raised under free grazing conditions (G2) had very low serum calcium values, and supplementation was unable to improve them. Meanwhile, phosphorus and magnesium values were below the required levels, but after supplementation (G1), they exceeded the minimum threshold. Mineral supplementation in the rearing of sheep in grazing systems is necessary during the entire production cycle, but it must be done taking into account the soil–plant–animal relationship, specifically for the Amazonian Region systems.

**Abstract:**

The objective of this study was to determine the effect of mineral supplementation on the serum concentration of calcium, phosphorus, and magnesium in pre- and postpartum Blackbelly sheep throughout three successive lambing periods under free grazing conditions in the Ecuadorian Amazon Region. The field work was carried out between January 2015 and February 2018 using 20 Blackbelly sheep belonging to the Centre for Research, Postgraduate Studies and Conservation of Amazon Biodiversity, Ecuador. The flock was randomly divided into two groups: Group 1 (G1) was fed with forage plus a supplementation (Pecutrin^®^ Mineral supplement plus vitamins A, D3, and E. Bayer HealthCare) and Group 2 (G2) was fed only with forage without mineral supplementation. Three blood samples from the coccygeal vein were taken from each sheep 30 days before lambing, 30 days after, and 60 days after lambing. Concerning the average of calcium, significant differences were found at different times inside each group and also between them (*p* < 0.0001 in both cases). As for the phosphorus, significant differences were found between the means of the groups for all times from 30 days after the second lambing season (*p* < 0.05). It was observed that the groups differed significantly in terms on the average of magnesium (considering a significance level of 0.05) 30 days before the first lambing and at all times measured from the 30 days after the second lambing (*p* < 0.005). In this study, we showed that Blackbelly sheep raised under free grazing conditions in the Ecuadorian Amazon Region had very low serum calcium values, and supplementation was unable to improve them. Meanwhile, phosphorus and magnesium levels were below the required values, but after supplementation, they exceeded the minimum threshold. Mineral supplementation in the rearing of sheep in grazing systems is necessary during the entire production cycle, but it must be done taking into account the soil–plant–animal relationship specifically for the Amazonian Region systems.

## 1. Introduction

The Blackbelly breed is of utmost relevance for meat production in the Ecuadorian Amazon Region, as it can adapt to extreme conditions. This fact is reflected in its low mortality, precocity and high reproductive fertility (multiple births), medium size, and productive longevity [1]. 

Many authors have shown that one of the problems regarding grazing ruminants is that pasture does not cover their protein, energy, or mineral needs, affecting the normal development of their metabolic processes [2], because their requirements depend exclusively on the forage’s composition [3]. The soils of the Amazonian Region, with a pH lower than 5.5 during most of the year, generate restrictions on plants. The soil acidity modifies physical and chemical properties. These negative effects are reflected by an excess of certain minerals—such as aluminum—and the deficiency of others, for instance, molybdenum. It should also be considered that forage resources have fluctuations throughout the year, both in quantity and in quality [4]. 

The intake of minerals in ruminants reared in grazing systems depends on the composition and the total consumption of forage, the consumption and the minerals content of water, and the composition of the soil [5]. Adequate amounts of essential minerals are critical to maximize the productivity and the health of livestock [6]. Furthermore, appropriate mineral levels improve the interaction between production and reproduction [7,8]. A widely used indicator to assess nutritional status, health, and well-being of sheep is the Body Condition Index, as indicated in the Animal Welfare Indicators (AWIN) protocols [9].

A suitable quantity and proportion of essential minerals prevents diseases by activating the immune system, improving growth, production, viability, and fertility [10]. Previously, some authors described the need for supplementation [11,12] and the importance of using concentrated mixtures for sheep according to age groups [13,14,15]. However, for many years, the supplementation under grazing conditions was not considered a cost-effective alternative. Preliminary studies showed that supplementation is profitable if kilograms of weaned lamb per female per year were analyzed. This variable involves the factors that most influence the profitability of the herd, since it considers prolificity of the female, milk production, maternal instinct, mortality, and weight gain of lambs [16].

The objective of this study was to determine the effect of mineral supplementation on the serum concentration of calcium, phosphorus, and magnesium in pre- and postpartum Blackbelly sheep throughout three successive lambing periods under free grazing conditions in the Ecuadorian Amazon Region. 

## 2. Materials and Methods

The field work was carried out in the Centre for Research, Postgraduate Studies and Conservation of Amazon Biodiversity (CIPCA). It is located in the Arosemena Tola canton, province of Napo, Ecuador (coordinates: 01°14.325′ S; 077°53.134′ W). The altitude varies between 580 and 990 m above sea level. The environment is tropical with an average annual rainfall of 4000 mm, an average relative humidity of 80%, and temperatures ranging between 15 and 25 °C.

The study was performed between January 2015 and February 2018. This interval included three lambing periods by the sheep under study. The same 20 Blackbelly females aged 24 to 32 months and with an average weight and standard deviation of 34 ± 4 kg were used.

The sheep were free to graze from 07:00 to 18:00, then housed overnight with water consumption ad libitum. They were randomly divided into two groups. Group 1 (G1) was fed with forage plus a supplement at a dose of 0.005 kg per animal per day every day from when the firstborn lamb was weaned until the end of the trial (Pecutrin^®^ Mineral supplement plus vitamins A, D3, and E. Bayer HealthCare; parts of the formula components: calcium min. 17% max. 20%; phosphorus min. 18%; magnesium min. 3.0%; relationship calcium–phosphorus 1.3:1) The mineral supplement dose was the minimum recommended by the manufacturer; each sheep had its own feeder space. Group 2 (G2) was fed only with forage without mineral supplementation. Table 1 shows the chemical composition of the predominant species in the pasturage used. The presence of aluminum and hydrogen ions determined by intense washing phenomena as a consequence of high rainfall generates acid soils with a pH of 5.2 [17]. This reduction in the pH affects chemical and biological characteristics of the soil, reducing the growth of plants. It also decreases the availability of nutrients such as calcium, magnesium, phosphorus, and potassium. In turn, acidic soils tend toward the accumulation of toxic elements for plants, such as aluminum and manganese [18].

The sanitary management routinely used for the sheep flock of CIPCA was applied. This includes deworming, baths to combat ticks and flies, vaccines for foot and mouth disease, alongside antifungal and antibacterial medicines.

Through the fodder mixture, the animals consumed 0.612 kg/day of dry matter (1.8% of their average weight). This percentage was estimated based on pastures advanced in their physiological maturity [21]. The contribution of Ca^2+^, P^3−^, and Mg^2+^ was 2.5 g/day, 0.5 g/day, and 1.8 g/day, respectively. Except for Mg^2+^, the mineral levels were below the daily minimum allowance recommended, given that the animals should consume 3.1 g/day, 2.9 g/day, and 1.0 g/day of Ca^2+^, P^3−^, and Mg^2+^, respectively [21]. Pecutrin^®^ (Bayer, Leverkusen, Alemania)., at the dose offered, contributed with a total of 0.6 g/day of Ca^2+,^ 0.5 g/day of P^3-^ and 0.9 g/day of Mg^2+^.

Blood samples were only taken from the female adult sheep. Three blood samples from the coccygeal vein were taken 30 days before lambing as well as 30 days and 60 days post lambing. Ten milliliters of blood was obtained from each animal in a vacuum tube without anticoagulant (BD Vacutainer^®^ red cap, Franklin Lakes, NJ, USA). The blood was centrifuged (3000 rpm × 15–30 min), and the separated serum was stored at −20 °C until analyzed.

Serum minerals were determined, including phosphorus (P^3−^) and magnesium (Mg^2+^). These two tests were performed by means of molecular spectrophotometry with the GENESYS 10 UV Series Thermo Scientific Spectrophotometer (Waltham, MA, USA). The reagents used were HUMAN (Germany), and the equipment was used according to its technical specifications for each of the samples. For calcium (Ca^2+^), we employed the AUDICOM, AC 9801 Electrolytic Analyzer (Maharashtra, India) with specific AUDICOM reagents according to the equipment’s technical specifications.

A descriptive statistical analysis was performed by calculating means. A linear regression model for correlated data was adjusted for inferential statistical analysis. This model took into account the correlation between the observations measured at the same unit over time by incorporating an intra-unit correlation structure. First, the correlation structure that best fit the data was chosen using the Akaike Penalized Likelihood Criterion. Then, we proceeded to interpret the results of the hypothesis tests for the model’s terms. If interaction between group and time was significant, contrasts were performed to compare if there were differences on average between the groups for each time. Statistical program R 3.6.3 was used.

## 3. Results

### 3.1. Calcium

There was not significant interaction between group and time for the calcium variable (*p* = 0.1469). There were significant differences between the average of calcium between times, and there were also significant differences between groups regarding the average of calcium (*p* < 0.0001 in both cases) (Table 2). Even though the model detected differences in the average calcium between groups, and the means were 2.44 mg/dL and 2.25 mg/dL, they were statistically different but not biologically.

### 3.2. Phosphorus

There was an interaction between group and time for the variable phosphorus (*p* = 0.0013). When performing contrasts to compare the means of the groups at each moment, significant differences were found between groups for all times from 30 days after the second lambing season (*p* < 0.05) (Table 3).

### 3.3. Magnesium

When adjusting the model, it was observed that there was interaction between group and time (*p* < 0.0001). The mean trajectory in time was not the same for both groups with respect to the variable Mg^2+^ (Table 4). When performing contrasts, it was observed that the groups differed significantly in terms of the average of magnesium (considering a significance level of 0.05) 30 days before the first lambing and at all times measured from the 30 days after the second lambing (*p* < 0.005).

## 4. Discussion

Few studies exist concerning feeding sheep raised in an Amazonian environment. Most regions have adapted their production systems according to knowledge obtained from production systems in temperate climates [22]. In the conditions of the Amazon Region, sheep grow consuming poor quality forage (that has excessive fiber, low protein, and low energy). The low quality of plants in acidic soils is due to the combination of toxicities of aluminum and manganese as well as deficiencies of sodium, phosphorus, potassium, calcium, magnesium, and some micronutrients, such as iron and zinc. Plants are subject to varying degrees of abiotic stress (soil acidity, mineral deficiencies and/or toxicities, droughts/floods, light/shadow quality, and extreme temperatures) and biotic stress (pests, diseases, and weeds). These tensions have an effect on growth and development, causing less absorption, a reduced utilization of absorbed nutrients and, consequently, a reduction in the efficient use of nutrients [23,24].

These characteristics could affect the normal development of metabolic and mineral processes in sheep. Blood is the most important bio-substrate for the estimation of an animal’s mineral state [25,26]. 

Minerals are essential nutrients for small ruminants and their concentrations in blood must oscillate within bounded intervals in order to maintain their health and well-being. Adequate concentrations of macronutrients allow structural, physiological, catalytic, and regulatory functions of the organism to be rightly developed [27,28].

Normal calcemic values in sheep are 11.5–12.8 mg/dL [29]. In this study, calcium levels in the ewes ranged between 2.6 and 2.8 mg/dL, well below the normal range. Quintero-Moreno [30] found values in sheep without supplementation of 8.73 mg/dL, while in supplemented sheep, they obtained values of 15.2 mg/dL. Underwood and Suttle [31] found that normal blood serum calcium values ranged from 7 to 8 mg/dL in young sheep, while Norton [32] described that calcium rarely is a limitation in fodder diets. The lower Ca^2+^ content in Amazonian pastures could be due to the natural dilution of the process by which the production of dry matter exceeds mineral uptake [33]. In this case, supplementation did not help to raise the calcium level; the calcium level in G1 increased (2.44 mg/dL) in comparison to G2 (2.25 mg/dL), thus they were statistically different but not biologically. This result could be explained in part because a minimum dose of 5 g/animal/day was used. However, the interactions with toxic minerals, which could interfere and decrease absorption, should also be considered. No placental retention problems were observed, nor ewes that fell after lambing, spontaneous fractures, or other related pathologies, although the values suggest acute subclinical hypocalcaemia. Herdt [34] suggested that animals would adapt to this condition and that such adaptation would be mainly based on glucose, non-esterified fatty acids (NEFA), and ketone body availability and supply. The improvement in Ca^2+^ levels could be reflected in the general state and surely in higher and better milk production, which should be seen in the weight of lamb weaned. Abarghani [35] found that Ca^2+^ and P^3−^ were deficient during different seasons in grazing animals in all regions and for all sheep categories. These authors concluded that, for sheep in continuous grazing, it was not possible to prevent deficiencies in the concentration of Ca^2+^ and P^3−^ in serum or plasma. They concluded that supplementing these small ruminants with a bioavailable mineral mixture could increase the blood level of these minerals. However, the authors stressed the importance of conducting more studies to determine requirements and economic benefits of mineral supplements.

Normal values of phosphatemia in sheep are 5–7.3 mg/dL [29]. In this study, the ewes’ phosphatemia values ranged between 4.03 and 6.71 mg/dL. In the first lambing, the values were lower than normal, but after incorporating the supplementation, the G1 values were within the normal values, as was observed for the G2 in the third lambing. Stojkovic [36] obtained similar results; he found that the phosphorus level in the blood serum of the sheep studied was between 3.84–4.61 mg/dL, which was below the normal limits necessary to meet the sheep’s needs for this macroelement. The low phosphorus content could be due to the low presence of P^3−^ in the acidic Amazonian soil and also to the low amount of this mineral in *Arachis pintoi* [23,24,37,38]. In ruminants, the amount of mineral excreted varies according to the type of diet. The amount and the source of phosphorus used in the diet also affect the kinetics of the mineral in the body [39,40]. The age of the animal can influence the use of dietary phosphorus, since it has been observed that young animals have higher absorption efficiency [41]. In this way, there are several aspects that can influence the phosphorus homeostasis in the organism and, therefore, the mineral’s kinetics. In recent years, the study of this mineral in the context of animal production has mainly focused on redefining the requirements of the mineral, seeking to minimize excretion without affecting animal performance [42]. In addition, the absorptions of calcium and phosphorus are independent of each other, which allow the sheep to adjust to different physiological demands [43]. The results showed an inverse Ca^2+^: P^3−^ ratio. For optimal performance in ruminants, it must be between 1.5:1 and 2.5:1 [26]. In our study, it was 1:1.7 at the beginning and 1:2.57 at the end, showing a clear mineral imbalance. However, in ruminants, the ratio of Ca^2+^ to P^3−^ only affects the absorption of these minerals if the diet is inadequate in said minerals [44]. The metabolism of Ca^2+^ is closely related to that of P^3−.^ The excess or the deficit of one mineral can affect the use of the other [44].

In this study, the serum levels of Mg^2+^ found in the sheep studied ranged between 2.5 and 2.7 mg/dL in the first lambing, even when the sheep of both groups were not supplemented. Supplementation allowed Mg^2+^ in blood to reach normal values (2.5–3.5 mg/dL) and to exceed it in the third lactation. The normal values of magnesemia in sheep are 5–7.3 mg/dL [29]. Our results coincided with those reported by Abarghani [35], with values that ranged between 2.9 and 3.7 mg/dL. The organic deficit of an important element such as Mg^2+^ results in less growth and development in the animal. McDowell [2] stated that magnesium is an enzymatic activator involved in the metabolism of carbohydrates and lipids, since it is a catalyst for a wide variety of enzymes. It is also part of protein synthesis through its action in ribosomal aggregation.

## 5. Conclusions

In this study, we showed that Blackbelly sheep raised in free grazing conditions in the Ecuadorian Amazon Region had very low calcium values in serum, and supplementation failed to improve them. Phosphorus and magnesium values were below the required values, but after supplementation, they exceeded the minimum threshold. Mineral supplementation in the rearing of sheep in grazing systems is necessary throughout the production cycle, but adjustments are essential upon taking into account the soil–plant–animal relationship for systems in the Amazon region.

## Figures and Tables

**Table 1:** Chemical composition of the pasture.

Pasturage	DM (kg/ha/yr)	Protein (%)	Calcium (%)	Phosphorus (%)	Magnesium (%)	IVD (%)
*Brachiaria decumbens*	17.585	10.6	0.20	0.18	0.15	44.4
*Arachis Pintoi*	6.212	19.4	1.7	0.21	0.63	59.2

DM: dry matter; IVD: in vitro digestibility. References: [19,20].

**Table 2:** Serum calcium levels throughout the three successive lambing seasons.

Ca^2+^ (mg/dL)	Lambing Season								
	First Lambing			Second Lambing			Third Lambing		
Days before or after	−30	+30	+60	−30	+30	+60	−30	+30	+60
Supplemented group	2.17	2.44	2.24	2.48	2.37	2.40	2.66	2.61	2.56
Not supplemented group	2.20	2.20	2.20	2.22	2.28	2.29	2.28	2.36	2.41

Results are expressed as mean values of calcium per days before or after lambing. No significant differences were observed between groups.

**Table 3:** Serum phosphorus levels throughout the three successive lambing seasons.

P^3−^ (mg/dL)	Lambing Season								
	First Lambing			Second Lambing			Third Lambing		
Days before or after	−30	+30	+60	−30	+30	+60	−30	+30	+60
Supplemented group	4.50	3.31	4.29	4.67	5.71	5.70	6.80	6.39	6.96
Not supplemented group	3.90	4.24	4.30	4.77	4.16	4.31	5.61	4.37	4.54
	ns	ns	ns	ns	*	ns	ns	*	*

Results are expressed as mean values of phosphorus per days before or after lambing. (*): significant differences (*p* < 0.001); (ns): no significant differences were observed between groups.

**Table 4:** Serum magnesium levels throughout the three successive lambing seasons.

Mg^2+^ (mg/dL)	Lambing Season								
	First Lambing			Second Lambing			Third Lambing		
Days before or after	−30	+30	+60	−30	+30	+60	−30	+30	+60
Supplemented group	2.18	2.42	2.25	2.70	3.45	4.50	3.49	3.95	3.86
Not supplemented group	2.72	2.90	2.54	2.17	2.39	2.29	2.36	2.50	2.73
	ns	ns	ns	ns	ns	*	*	*	*

Results are expressed as mean values magnesium per days before or after lambing. (*): significant differences (*p* < 0.001); (ns): no significant differences were observed between groups.
